# Association between HbA1c and red cell distribution width-standard deviation in newly diagnosed, treatment-naïve type 2 diabetes mellitus: a retrospective cross-sectional study

**DOI:** 10.1186/s12902-026-02405-9

**Published:** 2026-07-04

**Authors:** Kemal Ozan Lule, Anil Beyaz, Hamit Yildiz

**Affiliations:** 1 Department of Internal Medicine, Faculty of Medicine, Gaziantep University, Gaziantep, Turkey; 2 Department of Internal Medicine, Gaziantep City Hospital, Gaziantep, Turkey

**Keywords:** Type 2 diabetes mellitus, HbA1c, RDW-SD, Red cell distribution width, Glycemic status, Erythrocyte heterogeneity, Newly diagnosed diabetes, Treatment-naïve

## Abstract

**Background:**

Red cell distribution width-standard deviation (RDW-SD), a measure of erythrocyte volume heterogeneity, has attracted increasing interest as a hematologic marker linked to inflammation, oxidative stress, and metabolic dysfunction beyond anemia evaluation. However, evidence on the relationship between glycated hemoglobin (HbA1c) and RDW-SD in newly diagnosed, treatment-naïve type 2 diabetes mellitus (T2DM) remains limited. This study investigated the association between HbA1c and RDW-SD after accounting for hematologic, nutritional, renal, and metabolic factors that are routinely available at diagnosis.

**Methods:**

In this retrospective cross-sectional study, 202 adults aged 18–65 years with newly diagnosed, treatment-naïve T2DM were evaluated at a tertiary university hospital between September 2024 and January 2026. Patients with anemia, mean corpuscular volume abnormalities, advanced renal impairment, acute inflammation or infection, recent transfusion, prior antidiabetic therapy, active bleeding, or hematologic malignancy were excluded. Associations between HbA1c and RDW-SD were examined using correlation, partial correlation, and hierarchical linear regression analyses. Additional analyses assessed RDW-SD across HbA1c tertiles and tested the robustness of the association after inclusion of fasting glucose.

**Results:**

HbA1c showed a positive correlation with RDW-SD (*r* = 0.664, *p* < 0.001), which remained essentially unchanged after adjustment for age, sex, body mass index, hemoglobin, mean corpuscular volume, creatinine, ferritin, and vitamin B12 (partial *r* = 0.662, *p* < 0.001). Mean RDW-SD increased stepwise across ascending HbA1c tertiles (40.40 ± 3.04, 42.96 ± 3.38, and 46.13 ± 3.85 fL; p-trend < 0.001). In hierarchical regression, HbA1c produced the largest incremental gain in explained variance (ΔR² = 0.323, *p* < 0.001) and remained the strongest correlate of RDW-SD in the fully adjusted model (B = 1.535, β = 0.674, *p* < 0.001). In sensitivity analysis, the association persisted after additional adjustment for fasting glucose.

**Conclusions:**

In newly diagnosed, treatment-naïve T2DM, higher HbA1c levels were associated with greater RDW-SD. These findings are hypothesis-generating and do not establish RDW-SD as a clinical marker of glycemic burden or dysglycemia assessment.

**Clinical trial number:**

Not applicable.

**Supplementary Information:**

The online version contains supplementary material available at 10.1186/s12902-026-02405-9.

## Introduction

Type 2 diabetes mellitus (T2DM) is a chronic metabolic disease characterized by progressive hyperglycemia resulting from insulin resistance and relative beta-cell dysfunction. According to the 11th edition of the International Diabetes Federation (IDF) Diabetes Atlas, an estimated 589 million adults aged 20–79 years were living with diabetes worldwide in 2024, corresponding to a global prevalence of 11.1%, and this figure is projected to rise to 853 million by 2050 [1]. In addition to dysglycemia itself, T2DM is accompanied by a complex metabolic milieu that includes hyperglycemia-related oxidative stress, chronic low-grade inflammation, and advanced glycation end-product (AGE) formation, all of which are implicated in the development of diabetic complications [2, 3].

Glycated hemoglobin (HbA1c) remains the cornerstone biomarker for both the diagnosis and monitoring of glycemic status, reflecting average blood glucose over the preceding 8–12 weeks in relation to erythrocyte lifespan [4]. However, HbA1c values may also be influenced by conditions affecting erythrocyte turnover and red cell indices, including anemia, hemoglobinopathies, and chronic kidney disease [5]. This has led to growing interest in routinely available hematological parameters that may provide complementary information regarding the biological context of hyperglycemia in patients with diabetes.

Red cell distribution width (RDW), a routinely reported parameter in complete blood count analysis, reflects the heterogeneity of circulating erythrocyte volumes (anisocytosis). Although traditionally used in the differential diagnosis of anemia, RDW has increasingly been examined as a marker associated with systemic inflammation, oxidative stress, and adverse outcomes in various clinical settings, including cardiovascular disease, chronic kidney disease, and metabolic syndrome [6–8]. The standard deviation form of RDW (RDW-SD), which directly measures the width of the erythrocyte volume distribution curve in femtoliters, may offer analytical advantages over the coefficient of variation form (RDW-CV), as it is less dependent on mean corpuscular volume (MCV) and may more directly reflect erythrocyte volume heterogeneity [9]. For this reason, RDW-SD was selected as the primary erythrocyte heterogeneity index in the present study, whereas hemoglobin and MCV were used to define eligibility and were further considered as covariates because they reflect anemia status and average erythrocyte volume rather than volume dispersion.

Previous studies have reported associations between red cell distribution width and HbA1c in diabetic populations [10, 11]. However, the available literature remains limited by several important methodological issues. Many studies have included heterogeneous populations with varying diabetes duration, making it difficult to distinguish findings present at diagnosis from those related to longer disease exposure or treatment. In addition, most prior reports evaluated RDW-CV rather than RDW-SD and did not comprehensively account for major determinants of erythrocyte heterogeneity, such as anemia, nutritional deficiency, and renal dysfunction. Tsilingiris et al. [12] further suggested that the direction of the RDW–HbA1c relationship may vary according to glycemic status, demonstrating a positive association in non-diabetic individuals but an inverse association in patients with established T2DM. Taken together, these observations indicate that the relationship between HbA1c and RDW-related indices may differ across stages of dysglycemia and should be examined in clinically well-defined populations.

The metabolic milieu at the time of T2DM diagnosis also extends beyond glucose dysregulation. Atherogenic dyslipidemia, typically characterized by elevated triglycerides and reduced high-density lipoprotein cholesterol (HDL-C), commonly accompanies newly diagnosed T2DM and contributes to increased cardiovascular risk [13, 14]. The atherogenic index of plasma (AIP), calculated as log10(triglycerides/HDL-C), has been proposed as an integrated marker of this dyslipidemic profile and has been associated with insulin resistance, metabolic syndrome, and cardiovascular outcomes [15–17]. Whether this broader metabolic context is also reflected in erythrocyte volume heterogeneity at the time of diagnosis remains insufficiently characterized. RDW-SD is a routinely reported and low-cost parameter from standard complete blood count testing. Characterizing its association with HbA1c in newly diagnosed, treatment-naïve T2DM may inform future research on erythrocyte heterogeneity as part of the systemic hematologic context accompanying hyperglycemia at diagnosis.

Therefore, this retrospective cross-sectional study aimed to examine the association between HbA1c and erythrocyte volume heterogeneity, as assessed by RDW-SD, in a clinically well-defined cohort of newly diagnosed, treatment-naïve adults with T2DM using stringent exclusion criteria to minimize major confounders affecting erythrocyte indices. Secondary analyses evaluated the graded pattern of RDW-SD across HbA1c tertiles, the incremental contribution of HbA1c to RDW-SD variance in hierarchical regression models, and whether the observed association remained evident after additional consideration of fasting glucose.

## Methods

### Study design and setting

This retrospective cross-sectional study was conducted at the Department of Internal Medicine, Gaziantep University Şahinbey Research and Practice Hospital, Gaziantep, Türkiye. The study protocol was approved by the Gaziantep University Non-Interventional Clinical Research Ethics Committee (decision no. 2026/173; approval date: 04 March 2026). Approval for the use of retrospective hospital data was also obtained from the Chief Physician’s Office before data extraction. The study was conducted in accordance with the Declaration of Helsinki. Reporting was performed in line with the Strengthening the Reporting of Observational Studies in Epidemiology (STROBE) statement for cross-sectional studies.

### Study population

Medical records of adults with newly diagnosed T2DM who attended the General Internal Medicine outpatient clinics between 1 September 2024 and 15 January 2026 were reviewed. All consecutive patients meeting the inclusion criteria during the study period were identified through electronic medical record review and screened for eligibility. The patient screening and exclusion process is summarized in Supplementary Figure S1. Exclusions were applied hierarchically during eligibility assessment; once a record met an exclusion criterion, it was excluded at that step and was not further classified under subsequent criteria. Patients were eligible if they received a first diagnosis of T2DM at the index visit. T2DM was defined according to the American Diabetes Association Standards of Care in Diabetes [4] as HbA1c ≥ 6.5%. In this retrospective cohort, the diagnosis was established on the basis of the single index HbA1c measurement obtained during routine clinical evaluation consistent with T2DM, in accordance with usual outpatient practice; repeat confirmatory HbA1c testing was not systematically performed. The index visit was defined as the first visit at which the diagnosis was established.

All laboratory variables included in the analysis were obtained at the index visit as part of routine clinical care. HbA1c, RDW-SD, fasting glucose, ferritin, vitamin B12, creatinine, and all other laboratory variables included in the analysis were obtained from the same baseline/index-visit laboratory assessment. No research-specific tests were performed. For operational purposes, “newly diagnosed” was defined as receiving the first recorded diagnosis of T2DM at the index visit, with no documented previous diagnosis of diabetes in the medical records. “Treatment-naïve” was defined as having no prior use of antidiabetic medication before the index laboratory assessment, including metformin prescribed for insulin resistance or polycystic ovary syndrome.

### Eligibility criteria

Patients were eligible if they were aged 18–65 years and had a complete set of laboratory measurements obtained during the same index encounter. Required laboratory data comprised HbA1c, fasting glucose, complete blood count parameters including RDW-SD, hemoglobin, and MCV, lipid profile, ferritin, vitamin B12, C-reactive protein (CRP), creatinine, and estimated glomerular filtration rate (eGFR).

To minimize confounding from conditions known to affect erythrocyte indices, RDW-SD, and HbA1c interpretation, patients were excluded if they had anemia (hemoglobin < 13 g/dL in men or < 12 g/dL in women), erythrocyte volume abnormalities (MCV < 80 fL or > 100 fL), advanced renal impairment (eGFR < 30 mL/min/1.73 m²), or evidence of acute inflammation or infection (CRP > 10 mg/L or documented acute infectious process), because these conditions may alter erythrocyte turnover, erythrocyte volume distribution, inflammatory status, or glycation-related interpretation. Additional exclusion criteria were blood transfusion within the previous 3 months, prior use of antidiabetic therapy before the index assessment, including metformin prescribed for insulin resistance or polycystic ovary syndrome, hematological malignancy, and active bleeding, because these conditions or exposures may directly affect erythrocyte lifespan, hematologic indices, or the interpretation of baseline treatment-naïve status. Autoantibody testing for latent autoimmune diabetes in adults (LADA) was not part of routine evaluation during the study period; residual misclassification cannot be fully excluded.

### Data collection and definitions

Demographic data and anthropometric measurements were obtained from electronic medical records. BMI was calculated as weight in kilograms divided by height in meters squared. AIP was calculated as log₁₀(triglycerides/HDL-C) after conversion of triglyceride and HDL-C values from mg/dL to mmol/L [15]. eGFR was calculated using the 2021 Chronic Kidney Disease Epidemiology Collaboration (CKD-EPI) creatinine [18] and was used to operationalize the exclusion of advanced renal impairment, whereas serum creatinine was retained in descriptive and regression analyses as the routinely reported renal function marker. Fasting status was confirmed from the medical records, and only patients with documented fasting of at least 8 h before blood sampling were included in glucose and lipid analyses. All laboratory measurements were performed in the hospital’s central laboratory using standardized automated methods as part of routine clinical care. Complete blood count parameters were analyzed using the Sysmex XN-9000 hematology analyzer (Sysmex Corporation, Kobe, Japan), HbA1c was measured using the Lifotronic H9 Glycated Hemoglobin Analyzer (Lifotronic, Shenzhen, China). Result standardization is aligned with the DCCT and IFCC HbA1c reference methods, as recognized by the NGSP. Biochemical parameters were analyzed using the Beckman Coulter AU5800 clinical chemistry analyzer (Beckman Coulter, Inc., Brea, CA, USA).

### Statistical analysis

Continuous variables were assessed for normality using the Shapiro–Wilk test together with visual inspection of histograms and Q–Q plots. The distributional characteristics of the primary variables, RDW-SD and HbA1c, were specifically examined. No transformation was applied because visual inspection and model diagnostics did not indicate a distributional violation requiring transformation. Normally distributed variables are presented as mean ± standard deviation, whereas non-normally distributed variables are presented as median (interquartile range [IQR]). A complete-case analysis approach was used. Patients with missing values for any required clinical or laboratory variable were excluded from the analytic sample, and no statistical imputation was performed.

Associations between HbA1c and continuous variables were evaluated using Pearson correlation coefficients for normally distributed variables and Spearman’s rank correlation for non-normally distributed variables. Partial correlation analysis was performed to assess the HbA1c–RDW-SD association after adjustment for age, sex, BMI, hemoglobin, MCV, creatinine, ferritin, and vitamin B12.

For descriptive trend analysis, participants were grouped into sample-derived HbA1c tertiles to illustrate the graded pattern of RDW-SD across increasing HbA1c levels in a distribution-based manner, rather than to define clinically validated HbA1c risk categories. RDW-SD values across tertiles were compared using one-way analysis of variance, and linear trend across tertiles was assessed using linear regression with tertile category entered as an ordinal predictor. HbA1c was retained as a continuous variable in the primary correlation and hierarchical regression analyses. Clinically validated thresholds such as HbA1c 7% and 10% were not used for the primary subgroup stratification because the primary objective was to evaluate the graded distributional pattern of RDW-SD across increasing HbA1c levels within this newly diagnosed cohort, rather than to compare treatment-target or severe-hyperglycemia categories. To avoid information loss due to arbitrary categorization, HbA1c was retained as a continuous variable in the primary analyses, whereas HbA1c ≥ 10% was evaluated separately only in the exploratory ROC analysis.

Covariates for the hierarchical regression models were selected a priori based on biological plausibility, clinical relevance, and routine availability at the time of diagnosis, rather than by automated stepwise procedures or univariable p-value screening. The independent association between HbA1c and RDW-SD was examined using hierarchical linear regression with RDW-SD as the dependent variable. RDW-SD was modeled as the dependent variable because the primary analytical objective was to evaluate whether higher HbA1c levels were associated with greater erythrocyte volume heterogeneity at diagnosis. Model 1 included demographic and anthropometric covariates (age, sex, and BMI). Model 2 additionally included hematologic and nutritional variables relevant to erythrocyte indices (hemoglobin, MCV, ferritin, and vitamin B12). Model 3 further included metabolic and renal variables (AIP, LDL-C, and creatinine). Model 4 added HbA1c as the primary variable of interest. CRP was not entered into the primary regression models because acute inflammatory conditions had already been excluded a priori, resulting in a relatively narrow CRP range in the analytic sample; therefore, CRP was used as an eligibility-screening variable rather than as a covariate in the main models. Incremental model contribution was assessed using ΔR² and the F-change statistic. Multicollinearity was evaluated using variance inflation factors, with values > 5 considered indicative of problematic collinearity. Regression assumptions were assessed by visual inspection of residual histograms and normal probability plots. As a prespecified sensitivity analysis, the hierarchical regression was repeated with fasting glucose added to Model 3 to determine whether the HbA1c–RDW-SD association remained after accounting for a single fasting glucose measurement. As an additional exploratory analysis, the fully adjusted hierarchical regression model was repeated separately in male and female participants. An HbA1c × sex interaction term was also tested in the full sample to evaluate whether sex modified the association between HbA1c and RDW-SD. As an additional exploratory reverse-direction analysis, the model was specified with HbA1c as the dependent variable and RDW-SD as an independent variable, adjusted for the same covariates.

All analyses were performed using IBM SPSS Statistics, version 26.0 (IBM Corp., Armonk, NY, USA). All tests were two-sided, and *p* < 0.05 was considered statistically significant. The HbA1c–RDW-SD association was prespecified as the primary association of interest. The Benjamini-Hochberg(BH) procedure was applied to control the false discovery rate at 0.05 across the 16 secondary correlation analyses with HbA1c. Both raw and BH-adjusted p-values are reported in Table 1.

**Table 1:** Correlations of HbA1c with clinical and laboratory variables

Variable	*r* / ρ	*p*	*p* (BH-adjusted)
Age	0.017	0.815	0.876
BMI	0.102	0.149	0.264
AIP	0.470	< 0.001*	< 0.001*
RDW-SD	0.664	< 0.001*	< 0.001*
HDL-C	−0.392	< 0.001*	< 0.001*
LDL-C	−0.033	0.644	0.814
Creatinine	0.105	0.135	0.264
CRP	0.048	0.498	0.790
Hemoglobin	0.231	0.001*	0.003*
MCV	−0.031	0.658	0.814
Ferritin	0.000	0.997	1.000
Vitamin B12	0.043	0.540	0.790
Fasting Glucose	0.660	< 0.001*	< 0.001*
Triglycerides	0.183	0.009*	0.024*
AST	−0.117	0.098	0.222
ALT	−0.016	0.821	0.876

Pearson correlation coefficients are presented for approximately normally distributed variables, whereas Spear-man’s rho was used for non-normally distributed variables (fasting glucose, triglycerides, ferritin, vitamin B12, AST, and ALT). Abbreviations: BMI, body mass index; AIP, atherogenic index of plasma; RDW-SD, red cell dis-tribution width-standard deviation; HDL-C, high-density lipoprotein cholesterol; LDL-C, low-density lipoprotein cholesterol; CRP, C-reactive protein; MCV, mean corpuscular volume; AST, aspartate aminotransferase; ALT, alanine aminotransferase. Units for the listed variables are presented in Table 2. BH-adjusted p-values were computed using the Benjamini-Hochberg false discovery rate procedure across all 16 correlation tests. *p < 0.05

As an exploratory analysis, receiver operating characteristic curve analysis was performed to examine the discriminative ability of RDW-SD for identifying severe glycemic burden, defined as HbA1c ≥ 10%. This threshold was selected because HbA1c ≥ 10% is widely recognized in clinical practice guidelines as indicating marked hyperglycemia for which insulin initiation may be considered [4, 19]. The area under the curve was calculated with 95% confidence intervals. The optimal RDW-SD cut-off value was determined using the maximum Youden index (J = sensitivity + specificity − 1). This analysis was intended to be hypothesis-generating rather than a formal diagnostic accuracy evaluation.

### Sample size justification

To contextualize sample adequacy for the primary correlation of interest, the minimum required sample size was estimated based on the previously reported correlation between RDW and HbA1c (*r* = 0.32) [12]. Assuming a two-sided α of 0.05 and 80% power, approximately 74 participants would be required. Because multivariable regression models with several covariates were planned, a sample size of at least 180 was considered desirable to support model stability and more reliable covariate adjustment. The final sample of 202 exceeded this target.

## Results

### Study sample

A total of 202 patients with newly diagnosed T2DM were included in the final analysis. The mean age was 51.74 ± 8.31 years, and 123 participants (60.9%) were female. Mean BMI, HbA1c, and RDW-SD were 30.69 ± 5.06 kg/m², 8.62 ± 1.82%, and 43.17 ± 4.14 fL, respectively. Median fasting glucose and triglyceride levels were 170.50 mg/dL (Q1–Q3: 132.00–226.00) and 183.00 mg/dL (Q1–Q3: 125.75–266.25), respectively. Baseline demographic and laboratory characteristics are summarized in Table 2.

**Table 2:** Baseline demographic, anthropometric, and laboratory characteristics of the study population (*n* = 202)

Variable	Overall
Age, years	51.74 ± 8.31
Female, n (%)	123 (60.9)
BMI, kg/m²	30.69 ± 5.06
HbA1c, %	8.62 ± 1.82
AIP	0.28 ± 0.32
RDW-SD, fL	43.17 ± 4.14
HDL-C, mg/dL	46.50 ± 10.94
LDL-C, mg/dL	130.60 ± 34.50
Urea, mg/dL	31.11 ± 10.55
Creatinine, mg/dL	0.71 ± 0.16
CRP, mg/L	4.85 ± 3.05
Hemoglobin, g/dL	14.24 ± 1.46
MCV, fL	86.72 ± 3.60
Ferritin, ng/mL	31.40 (28.00–40.00)
Vitamin B12, pg/mL	266.00 (222.00–319.00)
Fasting Glucose, mg/dL	170.50 (132.00–226.00)
Triglycerides, mg/dL	183.00 (125.75–266.25)
AST, U/L	20.00 (16.00–24.00)
ALT, U/L	21.00 (17.00–29.25)

Data are presented as mean ± standard deviation, median (Q1–Q3), or n (%), as appropriate. Abbreviations: BMI, body mass index; HbA1c, glycated hemoglobin; AIP, atherogenic index of plasma; RDW-SD, red cell distribution width-standard deviation; HDL-C, high-density lipoprotein cholesterol; LDL-C, low-density lipoprotein cholesterol; CRP, C-reactive protein; MCV, mean corpuscular volume; AST, aspartate aminotransferase; ALT, alanine aminotransferase

### Correlation findings

Correlation analysis showed a strong positive association between HbA1c and RDW-SD (*r* = 0.664, *p* < 0.001). HbA1c was also positively correlated with AIP (*r* = 0.470, *p* < 0.001), hemoglobin (*r* = 0.231, *p* = 0.001), fasting glucose (ρ = 0.660, *p* < 0.001), and triglycerides (ρ = 0.183, *p* = 0.009), and negatively correlated with HDL-C (*r* = − 0.392, *p* < 0.001). No statistically significant correlations were observed between HbA1c and age, BMI, LDL-C, creatinine, CRP, MCV, ferritin, vitamin B12, AST, or ALT (Table 1). The association between HbA1c and RDW-SD remained significant after adjustment for age, sex, BMI, hemoglobin, MCV, creatinine, ferritin, and vitamin B12 in partial correlation analysis (partial *r* = 0.662, *p* < 0.001).

### RDW-SD across HbA1c tertiles

Mean RDW-SD values increased progressively across ascending HbA1c tertiles, from 40.40 ± 3.04 fL in the lowest tertile to 42.96 ± 3.38 fL in the middle tertile and 46.13 ± 3.85 fL in the highest tertile. The overall difference was statistically significant (ANOVA, F = 46.271, *p* < 0.001), and a significant positive linear trend was observed across tertiles (B = 2.865, β = 0.562, *p* < 0.001) (Table 3).

**Table 3:** RDW-SD values across sample-derived HbA1c tertiles

HbA1c tertile	HbA1c range, %	*n*	RDW-SD, fL
T1	6.5–7.4	66	40.40 ± 3.04
T2	7.5–9.0	69	42.96 ± 3.38
T3	9.1–14.3	67	46.13 ± 3.85

Data are presented as mean ± standard deviation. Overall group comparison was performed using one-way ANOVA, *p* < 0.001. Linear trend across tertiles: *p* < 0.001. P for trend was derived from linear regression using ordinal HbA1c tertiles (1–3) as the independent variable. RDW-SD, red cell distribution width-standard deviation; HbA1c, glycated hemoglobin

### Hierarchical regression analysis

In hierarchical linear regression analysis with RDW-SD as the dependent variable, the initial model including age, sex, and BMI was not significantly associated with RDW-SD (Model 1, adjusted R² = 0.023, *p* = 0.053). The addition of hemoglobin, MCV, ferritin, and vitamin B12 did not significantly improve model performance (Model 2, ΔR² = 0.034, *p* = 0.135). Incorporation of AIP, LDL-C, and creatinine significantly increased explanatory power (Model 3, ΔR² = 0.086, *p* < 0.001), with AIP emerging as a significant contributor (β = 0.311, *p* < 0.001).

**Fig. 1:**
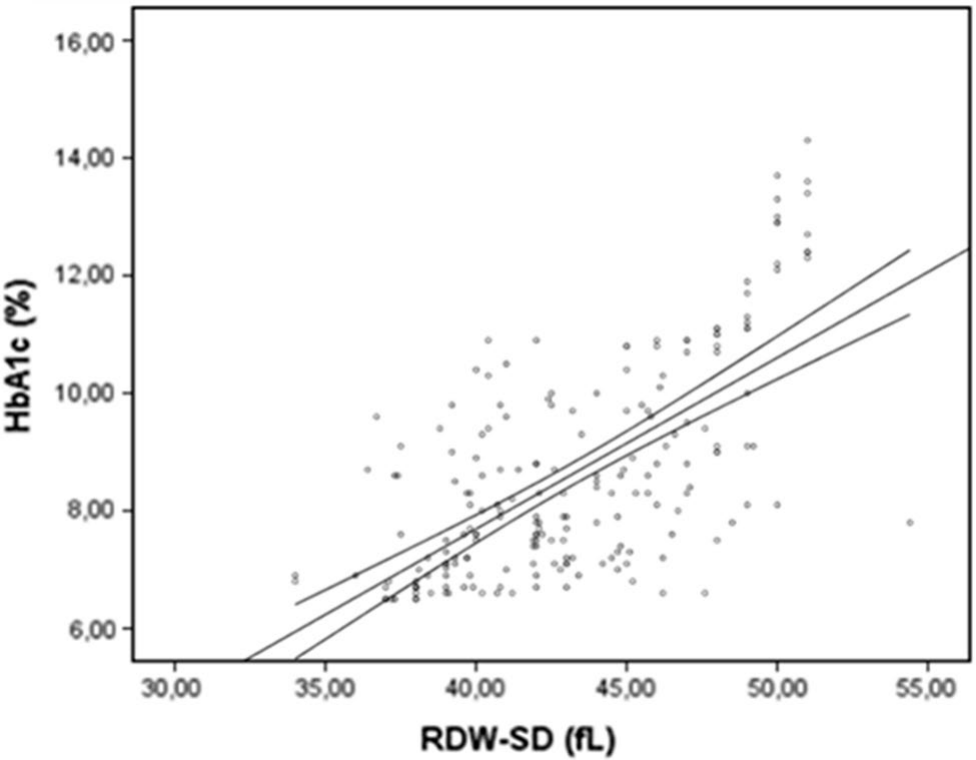
Association between HbA1c and RDW-SD in newly diagnosed, treatment-naïve T2DM. Scatter plot showing the relationship between HbA1c and RDW-SD in adults with newly diagnosed, treatment-naïve type 2 diabetes mellitus. The central line represents the fitted linear regression line, and the outer lines indicate the 95% confidence interval. Pearson correlation analysis showed a strong positive association between HbA1c and RDW-SD (*r* = 0.664, *p* < 0.001)

In the final model, the addition of HbA1c resulted in a substantial improvement in model fit (Model 4, ΔR² = 0.323, *p* < 0.001). In the fully adjusted model, HbA1c was the strongest correlate of RDW-SD (B = 1.535, β = 0.674, *p* < 0.001) and produced the largest incremental gain in explained variance. After multivariable adjustment, BMI also showed a statistically significant association with RDW-SD (β = 0.114, *p* = 0.041), although the effect size was modest. Vitamin B12 showed a weak but statistically significant negative association with RDW-SD (β = −0.128, *p* = 0.018), whereas ferritin did not show a statistically significant association in the fully adjusted model (β = 0.051, *p* = 0.355). AIP, which was significant in Model 3, was no longer statistically significant after the inclusion of HbA1c (β = −0.009, *p* = 0.882). Collinearity diagnostics indicated no meaningful multicollinearity in the final model, with VIF values ranging from 1.05 to 2.28 (Table 4). Visual inspection of residual histograms and normal probability plots indicated no major deviation from regression assumptions. Figure 1 illustrates the positive association between RDW-SD and HbA1c.

**Table 4:** Hierarchical linear regression analysis of variables associated with RDW-SD

Model	Variable	B	β	*p*	95% CI for B
**Model 1**	Age	0.029	0.058	0.412	−0.040 to 0.098
	Sex	−0.555	−0.066	0.362	−1.754 to 0.643
	BMI	0.158	0.192	0.008*	0.042 to 0.273
Adj R²=0.023, ΔR²=0.038, ΔF = 2.611, *p* = 0.053					
**Model 2**	Age	0.040	0.080	0.259	−0.030 to 0.109
	Sex	0.208	0.025	0.790	−1.330 to 1.747
	BMI	0.151	0.184	0.010*	0.036 to 0.266
	Hemoglobin	0.385	0.136	0.142	−0.130 to 0.901
	MCV	0.043	0.037	0.600	−0.118 to 0.204
	Ferritin	0.035	0.077	0.262	−0.026 to 0.096
	Vitamin B12	−0.007	−0.131	0.063	−0.014 to 0.000
Adj R²=0.039, ΔR²=0.034, ΔF = 1.778, *p* = 0.135					
**Model 3**	Age	0.019	0.037	0.602	−0.052 to 0.089
	Sex	1.019	0.120	0.225	−0.633 to 2.671
	BMI	0.098	0.119	0.090	−0.015 to 0.211
	Hemoglobin	0.382	0.135	0.126	−0.108 to 0.872
	MCV	0.105	0.091	0.187	−0.052 to 0.262
	Ferritin	0.029	0.063	0.341	−0.031 to 0.088
	Vitamin B12	−0.007	−0.129	0.052	−0.014 to 0.000
	AIP	4.078	0.311	< 0.001*	2.248 to 5.908
	LDL-C	−0.007	−0.055	0.432	−0.023 to 0.010
	Creatinine	1.589	0.061	0.452	−2.574 to 5.753
Adj R²=0.114, ΔR²=0.086, ΔF = 6.479, *p* < 0.001*					
**Model 4**	Age	0.015	0.029	0.604	−0.041 to 0.070
	Sex	−0.038	−0.005	0.955	−1.364 to 1.287
	BMI	0.093	0.114	0.041*	0.004 to 0.183
	Hemoglobin	−0.103	−0.037	0.610	−0.503 to 0.296
	MCV	0.101	0.087	0.112	−0.024 to 0.225
	Ferritin	0.023	0.051	0.355	−0.026 to 0.072
	Vitamin B12	−0.007	−0.128	0.018*	−0.012 to − 0.001
	AIP	−0.124	−0.009	0.882	−1.770 to 1.522
	LDL-C	0.005	0.045	0.427	−0.008 to 0.019
	Creatinine	0.054	0.002	0.974	−3.263 to 3.371
	HbA1c	1.535	0.674	< 0.001*	1.256 to 1.814

Adj R²=0.451, ΔR²=0.323, ΔF = 118.164, *p* < 0.001*

Dependent variable: RDW-SD. Model 1 included age, sex, and BMI. Model 2 additionally included hemoglobin, MCV, ferritin, and vitamin B12. Model 3 further included AIP, LDL-C, and creatinine. Model 4 additionally in-cluded HbA1c. B, unstandardized regression coefficient; β, standardized regression coefficient; CI, confidence interval; BMI, body mass index; MCV, mean corpuscular volume; AIP, atherogenic index of plasma; LDL-C, low-density lipoprotein cholesterol; HbA1c, glycated hemoglobin. Sex was coded as a binary variable with male as the reference category (male = 0, female = 1). *p < 0.05

### Exploratory ROC analysis

Because RDW-SD and HbA1c were measured concurrently, the ROC analysis evaluating RDW-SD for identifying HbA1c ≥ 10% was considered exploratory and is presented in Supplementary Figure S2.

### Sensitivity analysis

In the sensitivity analysis incorporating fasting glucose into Model 3, HbA1c remained associated with RDW-SD in the final model (B = 1.611, β = 0.707, *p* < 0.001; ΔR² = 0.221, F change = 78.696, *p* < 0.001), with acceptable collinearity (VIF = 2.27). Fasting glucose, which was a significant contributor to RDW-SD variance before HbA1c entry (β = 0.358, *p* < 0.001), became non-significant in the final model (β = −0.060, *p* = 0.430). BMI also retained a statistically significant association with RDW-SD (β = 0.112, *p* = 0.044). These findings suggest that RDW-SD showed a stronger cross-sectional association with HbA1c than with a single fasting glucose measurement (Supplementary Table 1).

### Exploratory sex-stratified and reverse-direction analysis

In exploratory sex-stratified fully adjusted regression analyses, HbA1c remained associated with RDW-SD in both men and women. The magnitude of the association was broadly comparable between male participants (B = 1.552, 95% CI: 1.129 to 1.975, β = 0.717, *p* < 0.001) and female participants (B = 1.528, 95% CI: 1.130 to 1.926, β = 0.648, *p* < 0.001). The fully adjusted model explained 52.6% of RDW-SD variance in men and 38.9% in women. In the full sample, the HbA1c × sex interaction term was not statistically significant (*p* = 0.72), suggesting no clear evidence that sex modified the HbA1c–RDW-SD association. These results are presented in Supplementary Table 2. In the reverse-direction model, RDW-SD remained independently associated with HbA1c after full covariate adjustment (B = 0.250, 95% CI: 0.204 to 0.295, β = 0.569, *p* < 0.001; adjusted R² = 0.536), supporting the consistency of the HbA1c–RDW-SD association in an exploratory reverse-direction model (Supplementary Table 3).

## Discussion

In this retrospective cross-sectional study of newly diagnosed, treatment-naïve patients with T2DM, HbA1c showed a positive association with RDW-SD. This association remained significant after adjustment for key covariates and was accompanied by a progressive increase in RDW-SD across ascending HbA1c tertiles. In hierarchical regression, the addition of HbA1c produced the largest incremental increase in explained variance, and HbA1c remained the strongest variable associated with RDW-SD in the fully adjusted model. Taken together, these findings suggest that higher HbA1c levels at diagnosis are associated with greater erythrocyte volume heterogeneity in this clinically selected cohort. The exploratory sex-stratified analyses further supported the consistency of this association, as HbA1c remained associated with RDW-SD in both men and women, and the HbA1c × sex interaction was not statistically significant. Although these subgroup findings should be interpreted cautiously because of the smaller sex-specific sample sizes, they do not suggest meaningful sex-based effect modification in the HbA1c–RDW-SD association.

These findings extend the existing literature in several respects. Previous studies have reported positive associations between RDW, most commonly RDW-CV, and glycemic status in diabetic populations, although the reported effect sizes have generally been modest [10, 20]. A weak but statistically significant positive correlation between RDW and HbA1c has previously been reported in adults with T2DM [10]. A positive association between RDW and HbA1c has also been described in non-diabetic individuals, together with an inverse relationship between RDW and fasting glucose, suggesting that the RDW–HbA1c relationship may not be explained solely by contemporaneous glycemia [20]. In comparison, the present study focused specifically on newly diagnosed, treatment-naïve T2DM, used RDW-SD rather than RDW-CV, and applied stringent exclusion criteria with additional adjustment for ferritin and vitamin B12. This narrower clinical framing may partly explain the stronger association observed in our cohort. The focus on RDW-SD was therefore deliberate, because RDW-SD quantifies the absolute dispersion of erythrocyte volumes, whereas MCV reflects average erythrocyte size and hemoglobin reflects anemia status. This distinction is relevant because the present analysis was designed to examine erythrocyte volume heterogeneity rather than anemia or mean cell volume.

A particularly relevant prior study suggested that the direction of the RDW–HbA1c relationship may vary according to glycemic status, being positive in non-diabetic individuals but inverse in established T2DM [12]. These findings raised the possibility that erythrocyte kinetics may differ across stages of dysglycemia. In that context, our results in newly diagnosed T2DM may indicate that a positive RDW–HbA1c relationship is still preserved at the time of diagnosis, before longer disease duration, treatment exposure, or more pronounced alterations in erythrocyte turnover potentially modify this pattern. This stage-specific perspective may help explain some of the heterogeneity across previous reports.

Several mechanisms may plausibly contribute to the observed association. Chronic hyperglycemia promotes non-enzymatic glycation of hemoglobin and erythrocyte membrane proteins, which may alter erythrocyte structure and rheological properties. Glycation-related changes involving Band 3 protein have been linked to impaired anion exchange, reduced deformability, and increased osmotic fragility [21]. In parallel, hyperglycemia is associated with oxidative stress through pathways involving AGE formation, the polyol pathway, and protein kinase C signaling [2, 3, 22]. These processes may contribute to membrane damage, altered erythrocyte survival, and broader heterogeneity in circulating cell volume. In addition, chronic low-grade inflammatory signaling may plausibly disturb erythropoietic dynamics and promote a mixed circulating erythrocyte population with greater variability in cell size. Supporting this biological framework, glycated erythrocytes have been shown to exhibit increased oxidative stress, greater phosphatidylserine exposure, and enhanced phagocytosis [23]. These mechanistic explanations are intended only as biologically plausible contextual interpretations; the present study did not measure AGE burden, oxidative stress markers, erythrocyte membrane alterations, or direct indices of erythrocyte turnover, and therefore cannot validate these pathways.

At the same time, the association between HbA1c and RDW-SD should not be interpreted as necessarily indicating a direct causal effect. Both parameters are linked, at least in part, to erythrocyte lifespan. HbA1c reflects cumulative glycation during erythrocyte survival, whereas RDW-SD reflects heterogeneity in erythrocyte volume distribution. If chronic hyperglycemia alters erythrocyte turnover, both measures could be influenced by shared upstream processes. The persistence of the association after adjustment for hemoglobin and MCV, together with the sensitivity analysis incorporating fasting glucose, argues against a purely artifactual explanation, but this possibility cannot be excluded in the absence of direct measures of erythrocyte turnover. Importantly, statistical adjustment for hemoglobin and MCV cannot fully isolate a truly independent biological signal between HbA1c and RDW-SD, because these parameters are mechanistically linked through erythrocyte volume, lifespan, and glycation kinetics. The use of multivariable models in the present study therefore reduces, but does not eliminate, the contribution of shared erythrocyte biology to the observed association. Residual confounding should also be considered. Although the models adjusted for hemoglobin, MCV, creatinine, ferritin, and vitamin B12, these variables do not fully capture erythrocyte turnover, chronic low-grade inflammatory activity, subclinical nutritional deficiencies, or other hematologic determinants of RDW-SD. Therefore, part of the observed association may still reflect unmeasured or incompletely measured hematologic and inflammatory factors. Conversely, RDW-SD may also be evaluated as a hematologic correlate of higher HbA1c levels in exploratory clinical models. However, because HbA1c and RDW-SD were measured concurrently and are both influenced by erythrocyte biology, neither direction of modeling can establish temporal or causal precedence.

The sensitivity analysis provides additional interpretive context. Fasting glucose was associated with RDW-SD before HbA1c was introduced into the model, but this association was no longer significant after HbA1c entry, whereas HbA1c remained associated with RDW-SD. This pattern suggests that RDW-SD showed a stronger cross-sectional association with HbA1c than with a single fasting glucose measurement. This interpretation remains hypothesis-generating, because the present design cannot determine whether RDW-SD reflects longer-term glycemic exposure or shared erythrocyte biology.

Another notable finding was the behavior of AIP across regression models. AIP was significantly associated with RDW-SD in Model 3, but this association was attenuated after inclusion of HbA1c in Model 4. This attenuation suggests that the RDW-SD association initially captured by AIP may partly reflect shared metabolic pathways linking insulin resistance, atherogenic dyslipidemia, and higher HbA1c levels, rather than an independent lipid-specific effect. Notably, variance inflation factors in the fully adjusted model were low (1.05–2.28), indicating that this attenuation reflects a shared metabolic substrate rather than statistical collinearity between AIP and HbA1c. This pattern is consistent with the close coexistence of dyslipidemia and hyperglycemia in newly diagnosed T2DM [13, 14]. It may also fit with recent evidence suggesting a non-linear relationship between HbA1c and AIP, with steeper deterioration in atherogenic lipid profile beyond intermediate HbA1c levels [24]. In our cohort, the mean HbA1c was 8.62%, placing many participants within a range in which higher HbA1c levels and atherogenic dyslipidemia may substantially overlap. Even so, this interpretation should remain cautious, because the present design does not allow temporal disentanglement of these metabolic processes.

BMI also remained significantly associated with RDW-SD in the fully adjusted model, although the effect size was modest. This finding may indicate that adiposity-related factors contribute to erythrocyte volume heterogeneity beyond the variables captured in the current models. In contrast, ferritin was not independently associated with RDW-SD, and the main HbA1c–RDW-SD association remained essentially unchanged after inclusion of ferritin and vitamin B12. This supports the interpretation that the primary association was not materially driven by the nutritional covariates available in this dataset. The weak inverse association observed between vitamin B12 and RDW-SD in the final model (β = −0.128) is biologically counter-intuitive, as B12 deficiency typically increases rather than decreases erythrocyte volume heterogeneity. Because patients with macrocytosis were excluded a priori, the range of vitamin B12 values in the present cohort was relatively narrow, and this finding likely reflects residual variance after MCV adjustment rather than a clinically meaningful effect. It is therefore reported as a hypothesis-generating observation only.

The weak inverse correlation between LDL-C and HbA1c was not statistically significant and should therefore not be overinterpreted. In T2DM, dyslipidemia is more characteristically defined by elevated triglycerides and reduced HDL-C than by isolated changes in LDL-C. In this respect, composite indices such as AIP may capture this metabolic pattern more effectively than LDL-C alone [14, 25].

The clinical implications of these findings should be stated cautiously. RDW-SD is an inexpensive and routinely available hematological parameter, and its association with HbA1c indicates that erythrocyte volume heterogeneity accompanies higher HbA1c levels at the time of diagnosis. However, the present results do not support RDW-SD as a substitute for HbA1c, nor do they establish prognostic utility. Rather than supporting clinical use at this stage, these findings identify RDW-SD as a hypothesis-generating hematological correlate of higher HbA1c in newly diagnosed, treatment-naïve T2DM patients without major hematologic, renal, or inflammatory confounders.

The exploratory ROC analysis should be interpreted within the same limits. Because RDW-SD and HbA1c were measured concurrently, the ROC findings do not establish diagnostic replacement or prospective discrimination. Instead, they indicate that greater erythrocyte volume heterogeneity clustered with HbA1c ≥ 10% in this sample. No inference regarding the use of RDW-SD as a substitute or surrogate for HbA1c testing is therefore drawn from these data.

### Strengths and limitations

This study has several strengths. First, it focused exclusively on newly diagnosed, treatment-naïve T2DM, thereby minimizing confounding related to antidiabetic treatment and variable disease duration. Second, stringent exclusion criteria were applied to reduce the influence of major non-diabetic determinants of RDW-SD, including anemia, MCV abnormalities, advanced renal impairment, acute inflammation, and recent transfusion. Third, ferritin and vitamin B12 were incorporated into the regression models, strengthening the assessment of whether the primary association persisted after accounting for available nutritional markers. Fourth, the sensitivity analysis including fasting glucose allowed a more nuanced comparison between longer-term and single-time-point glycemic measures. Fifth, RDW-SD, rather than RDW-CV, was used as the main erythrocyte heterogeneity parameter.

Several limitations should also be acknowledged. First, the retrospective cross-sectional design precludes causal inference and does not allow evaluation of temporal directionality between HbA1c and RDW-SD. In addition, T2DM diagnosis was based on a single index HbA1c measurement obtained during routine outpatient evaluation, and repeat confirmatory HbA1c testing was not systematically available. Therefore, potential diagnostic misclassification related to single-time-point HbA1c assessment cannot be fully excluded. Second, this was a single-center study from a tertiary university hospital and was restricted to adults aged 18–65 years, which may limit generalizability to other settings. In addition, regional ethnicity, referral patterns, outpatient attendance behavior, and local dietary-metabolic characteristics may differ from those of other populations, further limiting external validity. Third, the application of stringent exclusion criteria — including anemia, abnormal MCV, advanced renal impairment, acute inflammation, and recent transfusion — was necessary to minimize non-diabetic determinants of RDW-SD, but produced a hematologically homogeneous cohort that excludes precisely those clinical groups in whom an additional metabolic marker might be of greatest interest, namely patients with comorbidities that compromise HbA1c reliability. The present findings therefore apply primarily to newly diagnosed, treatment-naïve T2DM patients without these confounders, and should not be extrapolated to populations in whom HbA1c is itself unreliable. Importantly, these same conditions may also affect RDW-SD through altered erythrocyte turnover, inflammation, renal dysfunction, or hematologic abnormalities. Therefore, the current findings should not be interpreted as establishing RDW-SD as a complementary clinical marker in settings where HbA1c interpretation is compromised. Fourth, folate, transferrin saturation, reticulocyte count, and erythropoietin were not consistently available, restricting assessment of additional hematological mechanisms. Therefore, the present study cannot directly determine whether hyperglycemia influences RDW-SD through altered erythrocyte production or turnover. Fifth, because both HbA1c and RDW-SD are influenced by erythrocyte biology, some component of the observed association may reflect shared physiological substrate rather than a fully independent biological relationship. Therefore, the present data support only a cross-sectional HbA1c–RDW-SD association; mechanistic, temporal, prognostic, and clinical interpretations remain hypothesis-generating. Sixth, autoantibody testing for LADA was not routinely available; occult LADA cases may have been classified as T2DM. Because LADA may differ from typical T2DM in glycemic trajectory, insulin secretory reserve, and the broader metabolic and hematologic context, such residual misclassification could have influenced the magnitude of the observed HbA1c–RDW-SD association. In addition, the cohort showed a female predominance (60.9%), which likely reflects the higher utilization of outpatient general internal medicine services by women in our setting; sex was included as a covariate in all regression models and was not significantly associated with RDW-SD, suggesting that this demographic imbalance is unlikely to have materially influenced the primary findings. Although exploratory sex-stratified analyses and interaction testing were performed, these subgroup analyses should be interpreted cautiously because the sex-specific sample sizes were smaller than the full cohort. Finally, the ROC analysis was exploratory and internally derived, and the identified cut-off should therefore be regarded as preliminary.

## Conclusions

In this retrospective cross-sectional study of newly diagnosed, treatment-naïve patients with T2DM, HbA1c was associated with RDW-SD, and this relationship showed a graded pattern across HbA1c tertiles. The association remained evident after adjustment for demographic, hematological, nutritional, and metabolic variables, including ferritin and vitamin B12, and persisted after additional consideration of fasting glucose; however, because HbA1c and RDW-SD share underlying erythrocyte biology, statistical adjustment cannot establish full biological independence. HbA1c contributed the largest incremental increase in explained variance, whereas the association of fasting glucose was attenuated after HbA1c entry, suggesting that RDW-SD showed a stronger cross-sectional association with HbA1c than with a single fasting glucose measurement. These findings identify RDW-SD as a hematological parameter associated with higher HbA1c at diagnosis, but they do not establish RDW-SD as a marker of cumulative glycemic burden or as a complementary clinical tool. Prospective studies with direct measures of erythrocyte turnover, oxidative stress, and glycemic trajectories are needed to determine whether RDW-SD has independent biological or prognostic significance in newly diagnosed T2DM. 

## Supplementary Information

Below is the link to the electronic supplementary material.

Supplementary Material 1

Supplementary Material 2

Supplementary Material 3

Supplementary Material 4: Figure S1. Flow diagram of patient screening and exclusion. Flow diagram showing the identification, eligibility assessment, exclusion process, and final analytic sample of newly diagnosed T2DM patients. Exclusions were applied hierarchically during eligibility assessment; once a record met an exclusion criterion, it was excluded at that step and was not further classified under subsequent criteria. The final analytic sample included 202 newly diagnosed, treatment-naïve patients with type 2 diabetes mellitus

Supplementary Material 5: Figure S2. Exploratory receiver operating characteristic (ROC) curve of red cell distribution width-standard deviation (RDW-SD) for identifying HbA1c ≥ 10% in newly diagnosed, treatment-naïve type 2 diabetes mellitus (n = 202). Area under the curve = 0.871 (95% CI: 0.810–0.931, p < 0.001). The optimal cut-off value identified by the maximum Youden index (J = 0.619) was 44.95 fL (sensitivity 84.1%, specificity 77.8%). Because RDW-SD and HbA1c were measured concurrently in the same blood sample and were not assessed against an independent reference standard, this analysis is presented as a descriptive re-expression of the underlying correlation rather than as evidence of independent diagnostic utility

## Data Availability

The datasets analysed during the current study are not publicly available due to privacy and ethical restrictions related to the use of retrospective hospital records but are available from the corresponding author on reasonable request, subject to approval by the relevant institutional authorities.

## Abbreviations

AGE: Advanced glycation end-product
AIP: Atherogenic index of plasma
ALT: Alanine aminotransferase
AST: Aspartate aminotransferase
BMI: Body mass index
CKD-EPI: Chronic Kidney Disease Epidemiology Collaboration
CRP: C-reactive protein
eGFR: estimated glomerular filtration rate
HbA1c: Glycated hemoglobin
HDL-C: High-density lipoprotein cholesterol
IDF: International Diabetes Federation
LDL-C: Low-density lipoprotein cholesterol
LADA: Latent autoimmune diabetes in adults
MCV: Mean corpuscular volume
RDW: Red cell distribution width
RDW-CV: Red cell distribution width-coefficient of variation
RDW-SD: Red cell distribution width-standard deviation
STROBE: Strengthening the Reporting of Observational Studies in Epidemiology
T2DM: Type 2 diabetes mellitus
