# Supplementary material for: Association between HbA1c and red cell distribution width-standard deviation in newly diagnosed, treatment-naïve type 2 diabetes mellitus: a retrospective cross-sectional study
Source: BMC Endocr Disord. 2026 Jul 4;26:226. doi: 10.1186/s12902-026-02405-9 (PMC13445724; doi:10.1186/s12902-026-02405-9)
Supplement: Supplementary file 1 [file 12902_2026_2405_MOESM1_ESM.docx]

**Supplementary Table 1. Sensitivity analysis: Hierarchical regression with fasting glucose (dependent variable: RDW-SD)**

| **Model** | **Variables added** | **Adj R²** | **ΔR²** | **F change** | **p** |
| --- | --- | --- | --- | --- | --- |
| **Model 1** | Age, sex, BMI | 0.023 | 0.038 | 2.611 | 0.053 |
| **Model 2** | Hemoglobin, MCV, ferritin, vitamin B12 | 0.032 | 0.018 | 1.889 | 0.154 |
| **Model 3** | AIP, LDL-C, creatinine, fasting glucose | 0.209 | 0.188 | 11.928 | <0.001* |
| **Model 4** | HbA1c | 0.437 | 0.221 | 78.696 | <0.001* |

In the final model, HbA1c (β = 0.707, *p* < 0.001, VIF = 2.27) and BMI (β = 0.112, *p* = 0.044) were independently associated with RDW-SD. Fasting glucose lost its independent association after HbA1c entry (β = −0.060, *p* = 0.430). Adj R², adjusted coefficient of determination; ΔR², change in R²; β, standardized regression coefficient; VIF, variance inflation factor; BMI, body mass index; MCV, mean corpuscular volume; AIP, atherogenic index of plasma; LDL-C, low-density lipoprotein cholesterol; HbA1c, glycated hemoglobin; RDW-SD, red cell distribution width–standard deviation. Sex was coded as a binary variable with male as the reference category (male = 0, female = 1). *p < 0.05.
