# Supplementary material for: Association between HbA1c and red cell distribution width-standard deviation in newly diagnosed, treatment-naïve type 2 diabetes mellitus: a retrospective cross-sectional study
Source: BMC Endocr Disord. 2026 Jul 4;26:226. doi: 10.1186/s12902-026-02405-9 (PMC13445724; doi:10.1186/s12902-026-02405-9)
Supplement: Supplementary file 2 [file 12902_2026_2405_MOESM2_ESM.docx]

**Supplementary Table 2. Sex-stratified fully adjusted linear regression analysis of variables associated with RDW-SD**

| **Variable** | **Male (n = 79)**  **B (95% CI)** | **β** | **p** | **Female (n = 123)**  **B (95% CI)** | **β** | **p** |
| --- | --- | --- | --- | --- | --- | --- |
| Age | 0.059 (−0.024, 0.141) | 0.125 | 0.162 | 0.011 (−0.070, 0.091) | 0.020 | 0.795 |
| BMI | 0.121 (−0.019, 0.261) | 0.144 | 0.090 | 0.083 (−0.037, 0.204) | 0.100 | 0.172 |
| Hemoglobin | −0.072 (−0.662, 0.519) | −0.023 | 0.809 | −0.091 (−0.727, 0.544) | −0.023 | 0.777 |
| MCV | 0.110 (−0.056, 0.277) | 0.112 | 0.191 | 0.058 (−0.137, 0.252) | 0.044 | 0.557 |
| Ferritin | 0.055 (−0.007, 0.118) | 0.145 | 0.083 | −0.009 (−0.093, 0.074) | −0.017 | 0.829 |
| Vitamin B12 | −0.005 (−0.014, 0.004) | −0.095 | 0.269 | −0.007 (−0.015, 0.001) | −0.142 | 0.066 |
| AIP | −0.217 (−2.440, 2.006) | −0.018 | 0.846 | −0.466 (−2.930, 1.999) | −0.033 | 0.709 |
| LDL-C | −0.001 (−0.022, 0.019) | −0.013 | 0.890 | 0.011 (−0.009, 0.030) | 0.083 | 0.286 |
| Creatinine | −2.167 (−7.204, 2.870) | −0.078 | 0.394 | 0.165 (−4.440, 4.770) | 0.005 | 0.943 |
| HbA1c | 1.552 (1.129, 1.975) | 0.717 | <0.001 | 1.528 (1.130, 1.926) | 0.648 | <0.001 |
| **Model adjusted R²** | 0.526 | | | 0.389 | | |

The model corresponds to the fully adjusted model used in the hierarchical regression framework. B, unstandardized regression coefficient; CI, confidence interval; β, standardized regression coefficient. The dependent variable was RDW-SD. The fully adjusted model included age, BMI, hemoglobin, MCV, ferritin, vitamin B12, AIP, LDL-C, creatinine, and HbA1c. The HbA1c × sex interaction term was tested in the full sample and was not statistically significant (p = 0.72). Abbreviations: BMI, body mass index; MCV, mean corpuscular volume; AIP, atherogenic index of plasma; LDL-C, low-density lipoprotein cholesterol; HbA1c, glycated hemoglobin; RDW-SD, red cell distribution width-standard deviation.
