# Supplementary material for: Association between HbA1c and red cell distribution width-standard deviation in newly diagnosed, treatment-naïve type 2 diabetes mellitus: a retrospective cross-sectional study
Source: BMC Endocr Disord. 2026 Jul 4;26:226. doi: 10.1186/s12902-026-02405-9 (PMC13445724; doi:10.1186/s12902-026-02405-9)
Supplement: Supplementary file 3 [file 12902_2026_2405_MOESM3_ESM.docx]

**Supplementary Table 3. Reverse-direction multivariable linear regression with HbA1c as the dependent variable**

| **Variable** | **B (95% CI)** | **β** | **p** |
| --- | --- | --- | --- |
| Age | −0.005 (−0.028, 0.017) | −0.024 | 0.646 |
| Sex | 0.500 (−0.028, 1.028) | 0.134 | 0.063 |
| BMI | −0.024 (−0.060, 0.012) | −0.067 | 0.193 |
| Hemoglobin | 0.259 (0.100, 0.419) | 0.209 | 0.002 |
| MCV | −0.023 (−0.073, 0.027) | −0.045 | 0.368 |
| Ferritin | −0.016 (−0.036, 0.004) | −0.079 | 0.116 |
| Vitamin B12 | 0.002 (−0.001, 0.004) | 0.075 | 0.132 |
| AIP | 1.785 (1.174, 2.395) | 0.310 | <0.001 |
| LDL-C | −0.006 (−0.011, −0.001) | −0.113 | 0.028 |
| Creatinine | 0.753 (−0.572, 2.079) | 0.066 | 0.264 |
| RDW-SD | 0.250 (0.204, 0.295) | 0.569 | <0.001 |
| **Model adjusted R²** | 0.536 | | |

B, unstandardized regression coefficient; CI, confidence interval; β, standardized regression coefficient. The dependent variable was HbA1c. The model included age, sex, BMI, hemoglobin, MCV, ferritin, vitamin B12, AIP, LDL-C, creatinine, and RDW-SD, corresponding to the same covariate set as the primary fully adjusted model. Sex was coded as a binary variable with male as the reference category. Abbreviations: BMI, body mass index; MCV, mean corpuscular volume; AIP, atherogenic index of plasma; LDL-C, low-density lipoprotein cholesterol; HbA1c, glycated hemoglobin; RDW-SD, red cell distribution width-standard deviation.
